# Prof. J. B. Beck’s Observations on the Signs of Live and Still Birth

**Published:** 1842-04-16

**Authors:** 


### BIBLIOGRAPHICAL NOTICES.

*Observations on some of the Signs of Live and Still Birth, in their application to Medical Jurisprudence.* By John B. Beck, M. D., Prof. of Medical Jurisprudence in the College of Physicians of the University of the State of N. York. 8vo. pp. 8.

This pamphlet presents the results arrived at by Prof. Beck from the examination of ten *neonati* with reference to the question—what are the tests to be relied on determining the fact of respiration's having taken place in an infant? We give an abstract of the author's conclusions. He separately considers the three most important tests resorted to in determining the question. 1. The static test. 2. The hydrostatic test. 3. The state of the ductus arteriosus.

1. *The static test*, founded on the fact, that the act of respiration causes an increase in the weight of the lungs. There are two forms in which this test has been applied. The first is by comparing the weight of the lungs with that of the body. This is commonly called Ploucquet's test. The second is that of taking the absolute weight of the lungs.

*a. Ploucquet's test*, so called from its having been originally suggested by Ploucquet, is founded on the fact, that as soon as respiration takes place in the new-born infant, an additional quantity of blood penetrates the lungs, in consequence of which, these organs become heavier than anterior to respiration. As the weight of the body of the child cannot undergo any change, he suggested, accordingly, that a comparison of the weight of the body of the child with the weight of its lungs, would furnish a test by which to determine whether it had respired or not. From the few observations which he made, he came to the conclusion that where respiration had not taken place, the proportion between the weight of the lungs and that of the body, was as 1 to 70; while on the other hand, where respiration had taken place, it was as 1 to 35; or in other words, that the weight of the lungs was doubled in consequence of respiration. In the ten cases which I have examined, says Dr. Beck, the proportions are the following:—Of the children that had respired, the average proportion was 1 : 40. Of those that had not respired, the average proportion was 1 : 47.

The conclusions to be drawn from these observations, are manifestly adverse to the accuracy of this test. Neither the individual cases, nor the general averages, correspond with the proportions suggested by Ploucquet. It may be asked, then, is this test to be rejected altogether? As an infallible one, it certainly should be. Notwithstanding this, it is still, I think, valuable as furnishing corroborative proof, and should, therefore, never be neglected. It should always be taken in connection with the other signs; and when this is done, it may aid very materially in coming to a correct conclusion.

*b. Absolute weight of the lungs.* By some it has been supposed, that the actual weight of the lungs would furnish another criterion of the fact of respiration having taken place or not. Accordingly, an average weight of 1000 grains has been proposed for the lungs of a child which has respired, and 600 grains for those of a child which has not respired. This test is, however, still more uncertain than that of Ploucquet.

An analysis of the weights in the cases examined by me shows how fallacious it must be. In three cases, before respiration took place, the lungs weighed more than in those which had respired; while the general average weight was greater in those which had not respired—just the reverse of what it ought to be according to this test.

2. *The hydrostatic test.* This test is founded upon the difference in the specific gravity of the lungs before and after respiration. Every observation which I have been enabled to make, has confirmed me in the general accuracy of this test. It is liable, however, to certain fallacies or objections which require to be understood, to enable us to make a correct practical application of the test. On the one hand, lungs which *have not respired* may float from *putrefaction*—from *artificial inflation*—from *emphysema*; while, on the other hand, lungs which *have respired* may sink from *disease*, or from the respiration *being feeble or imperfect*. Of these I shall only notice two, as they are the only ones, of which illustrations have occurred in the cases which I have examined. They are, however, the most important of all the objections.

*a. Putrefaction.* That the lungs of a child which has not respired may float in consequence of putrefaction, is beyond doubt. The modes of distinguishing it from the floating of respiration are simple and obvious. *a.* By the air bubbles being visible under the external covering of the lungs. In vital respiration this is not the case. *b.* By the ease with which the air can be pressed out of the lungs. By simply squeezing them in the hand, they can readily be made to sink in water. In vital respiration this cannot be done. *c.* By the sinking of the internal portion of the lungs. The air, in putrefaction, forms on the surface of the lungs; and hence the internal part, if cut out and put into water, will not float. In vital respiration, the internal part will float more readily than the external part of the lungs.

Dr. Beck gives a case illustrating, very strikingly, the fact that the lungs of a still born child may float from putrefaction, and at the same time confirming the accuracy of the tests, by which it may be distinguished from the floating which is the result of vital respiration.

*b. Artificial inflation.* That the lungs of a child which has not respired may be artificially inflated, so as to cause them to float, is well established; and when this is the case, it presents one of the most puzzling problems—to distinguish it from vital respiration. The only test upon which any reliance can be placed, is the application of suitable pressure to the lungs. If the floating be the result of vital respiration, no degree of pressure can expel the air from the lungs sufficiently to cause them to sink; while, on the other hand, in cases of artificial inflation, this can be done.

Dr. Beck reports an exceedingly interesting case, illustrating the effects of artificial inflation, and showing how nearly they resemble those of vital respiration. The floating of the lungs was almost perfect, and the weight of the lungs (900 grains) was nearly that of the usual average standard of children that have respired. On the other hand, the sinking of the lungs, after due pressure, the relative weight of the lungs and the body, 1 : 52, and the state of the ductus arteriosus, were in favour of artificial inflation.

3. *State of the ductus arteriosus*, also called the *Vienna* test, from its being originally suggested by Prof. Bernt, of Vienna. It is founded on certain changes, which take place in the ductus arteriosus, immediately after respiration. In the mature fœtus before respiration, this duct is about half an inch long, cylindrical in shape, with a diameter about equal to that of the pulmonary artery, and more than double the size of the branches of that artery, each of which is equal to that of a crow quill. If the child have respired a few moments, the duct becomes conical in shape, with its contracted part towards the aorta. If the child have respired for some hours or a day, it becomes cylindrical again in shape, but lessened in length and diameter.

It is much less now than the pulmonary artery, and not larger than the branches of that artery. If the child have respired for several days or a week, the duct will be found still more contracted; its diameter will be not larger than a crow quill, while the branches of the pulmonary artery are much enlarged to the size of a goose quill. The results of my cases go strongly to support the accuracy of these observations; but it is to be recollected that the test is not to be relied upon in all cases. This has been shown particularly by Orfila.

The results of these observations of Dr. Beck's on a subject so practically important, it is scarcely necessary to say, are of great value.

